# Low Overpotential Amperometric Sensor Using Yb_2_O_3_.CuO@rGO Nanocomposite for Sensitive Detection of Ascorbic Acid in Real Samples

**DOI:** 10.3390/bios13060588

**Published:** 2023-05-29

**Authors:** Jahir Ahmed, Mohd Faisal, Jari S. Algethami, Mabkhoot A. Alsaiari, Saeed A. Alsareii, Farid A. Harraz

**Affiliations:** 1 Promising Centre for Sensors and Electronic Devices (PCSED), Advanced Materials and Nano-Research Centre, Najran University, Najran 11001, Saudi Arabia; janurulislam@nu.edu.sa (J.A.); mfahsan@nu.edu.sa (M.F.); jsalgethami@nu.edu.sa (J.S.A.); mamalsaiari@nu.edu.sa (M.A.A.); alsareii@nu.edu.sa (S.A.A.); 2 Department of Chemistry, Faculty of Science and Arts, Najran University, Najran 11001, Saudi Arabia; 3 Department of Chemistry, Faculty of Science and Arts at Sharurah, Najran University, Sharurah 68342, Saudi Arabia; 4 Department of Surgery, College of Medicine, Najran University, Najran 11001, Saudi Arabia

**Keywords:** ascorbic acid, amperometric sensor, Yb_2_O_3_.CuO@rGO, vitamin C, human blood serum

## Abstract

The ultimate objective of this research work is to design a sensitive and selective electrochemical sensor for the efficient detection of ascorbic acid (AA), a vital antioxidant found in blood serum that may serve as a biomarker for oxidative stress. To achieve this, we utilized a novel Yb_2_O_3_.CuO@rGO nanocomposite (NC) as the active material to modify the glassy carbon working electrode (GCE). The structural properties and morphological characteristics of the Yb_2_O_3_.CuO@rGO NC were investigated using various techniques to ensure their suitability for the sensor. The resulting sensor electrode was able to detect a broad range of AA concentrations (0.5–1571 µM) in neutral phosphate buffer solution, with a high sensitivity of 0.4341 µAµM^−1^cm^−2^ and a reasonable detection limit of 0.062 µM. The sensor’s great sensitivity and selectivity allowed it to accurately determine the levels of AA in human blood serum and commercial vitamin C tablets. It demonstrated high levels of reproducibility, repeatability, and stability, making it a reliable and robust sensor for the measurement of AA at low overpotential. Overall, the Yb_2_O_3_.CuO@rGO/GCE sensor showed great potential in detecting AA from real samples.

## 1. Introduction

Ascorbic acid (AA) is a vital biomolecule that is present in a variety of naturally occurring sources, including fruits and vegetables, and that functions as a nutrient and antioxidant [1]. It is essential to numerous bodily metabolic activities including activating the immune system, aiding in wound healing, helping with the absorption of iron, and protecting against damage to bones and teeth [2]. Additionally, AA serves as a cofactor during the synthesis of collagen and carnitine [3]. In addition, AA has been demonstrated to provide protective effects against oxidative illnesses such as heart disease, several cancers, AIDS, the common cold, etc. [4]. However, there is no AA produced by the human body and it can only be obtained through the consumption of foods and medicines [5]. AA is a crucial ingredient in dietary and pharmaceutical supplements [6]. Human blood serum typically contains between 28.5 and 85.2 µM of AA, and the amount of AA in blood serum can provide information about a person’s general state of health [7]. Scurvy and anemia can result from an AA deficit in blood serum, while an excess of AA can lead to gastric irritation or diarrhea [8]. Therefore, it is crucial to have precise and efficient techniques for figuring out how much AA is present in foods, medications, and blood serum.

Direct titration [9], chromatography [10], spectrophotometry [11], and solid-phase spectrophotometry [12] are currently used methods for AA measurement. However, such methods are costly and require skilled personnel as well as challenging analytical measures for multi-sample preparation. To address these issues, researchers are working to develop efficient and cost-effective methods for real-time and in situ AA determination. The advantages of electrochemical detection include quicker measurements, reduced sample size, reduced costs, and an absence of pre-concentration processes, making them handy, portable, and simple to use with miniaturized electrodes [13]. However, due to its irreversible nature and high overpotential requirements, the electrochemical AA oxidation at a bare electrode might have negative effects on selectivity, electrode fouling, and repeatability. Therefore, it is indispensable to fabricate an electrode surface that enables efficient AA detection with less overpotential. In recent years, to detect AA, researchers have proposed several sensors, including electrodes modified with metals and metal oxides [14], alkylimidazolium salt [15], graphene derivatives [16], carbon nanotubes [14], and polymers [17].

Due to their potential technological uses and intriguing optical and structural characteristics, semiconducting doped nanostructured materials comprising transition metal oxides have drawn a lot of attention. Due to their size, shape, and surface, these materials have distinct physical and chemical properties that make them relevant in a variety of study fields and applications for industry. In particular, metal oxide-based sensors have been explored for their diverse uses in areas such as the protection of the environment, chemical process management, personal security, healthcare, and in the military [18,19,20]. These sensors have several advantages, including their compact size, affordable price, lower power consumption, straightforward processing, and good stability [21]. Previously, researchers have investigated various types of metal oxides, such as CuO [22,23], MnO_2_ [24], NiO [25], Fe_2_O_3_ [26], and ZnO [27], as electron mediators for sensing applications. Additionally, doped metal oxides, such as NiO.CoO nanocomposites [28], CdO.SnO_2_.V_2_O_5_ [29], CuO.In_2_O_3_ [30], CuO.Nd_2_O_5_ [31], CuO.NiO [32], and CuO.ZnO [33], have been studied as efficient sensing materials with higher sensitivity, small detection limits, wide linear dynamic ranges, and quick response times. CuO, a *p*-type semiconductor, has shown particularly good performance as an electrocatalyst in sensing applications [34]. To improve the performance of CuO, researchers have also investigated using other semiconductor metal oxides such as In_2_O_3_ [30], Nd_2_O_5_ [31], NiO [32], TiO_2_ [35], and SnO_2_ [36] in combination with CuO as bimetallic oxide pairs. Ytterbium oxide (Yb_2_O_3_) has also been explored for use in sensing applications [37,38,39]. Yb_2_O_3_ is a lanthanide-based C-type sesquioxide that can exhibit electrochemical redox characteristics [40]. In addition, the electrical as well as structural and morphological properties of the CuO can be engineered through doping of the CuO matrix with suitable dopants. Many trivalent lanthanide ions and their oxides have been reported as excellent dopants for CuO with modified and enhanced electrical properties. However, a detailed literature survey revealed very few reports for the synthesis of Yb_2_O_3_-doped CuO structures. Thus, here we have synthesized the Yb_2_O_3_-doped CuO nanostructures through the coprecipitation method. Furthermore, this Yb_2_O_3_.CuO was combined with rGO to obtain the ternary Yb_2_O_3_.CuO@rGO nanocomposite for the AA sensing with improved sensor parameters.

A lot of research has been carried out on the applicability of carbonaceous nanomaterials for sensing applications, including reduced graphene oxide, activated carbon, mesoporous carbon, and carbon nanotubes. In particular, graphene, a sheet of sp^2^-bonded carbon atoms, with a particular surface area, low density, outstanding electrical conductivity, and great mechanical properties, has drawn a lot of interest [41,42]. Graphene-based 3D nanomaterials have also generated huge interest due to their high surface area, lower density, better electrical conductivity, and exceptional mechanical properties [43,44]. Composite materials made of metal oxides and graphene have recently been explored for their stability, long-term storage, and photo-catalytic capabilities [45,46]. Many graphene-based nanomaterials have been studied in sensing applications [47,48,49]. However, rare earth oxide-transition metal oxide-reduced graphene nanocomposite has hardly been studied in sensing applications. Hence, in this work, we developed and examined Yb_2_O_3_.CuO@rGO nanocomposite (NC) as the active sensing material for AA detection.

Inspired by previous works available in the literature, we synthesized Yb_2_O_3_-doped CuO nanoparticles to improve stability, sensitivity, and selectivity, and then used a simple sonication technique to synthesize the Yb_2_O_3_.CuO@rGO NC sensing material. This study presents a simple method for preparing an electrochemical AA sensor using Yb_2_O_3_.CuO@rGO NC that offered improved selectivity and sensitivity. As far as we are aware, this will be the maiden article utilizing Yb_2_O_3_.CuO@rGO NC to develop an enzyme-less AA electrochemical sensor.

## 2. Materials and Methods

### 2.1. Materials

All necessary chemicals, including ascorbic acid, copper (II) nitrate, ytterbium (III) nitrate, sodium hydroxide, reduced graphene oxide, NaH_2_PO_4_, Na_2_HPO_4_, citric acid, glucose, uric acid, dopamine, sodium chloride, and calcium nitrate, were purchased from Sigma–Aldrich, and utilized exactly as they were given. All solutions were made using double-distilled water. The XPS investigation of Yb_2_O_3_.CuO@rGO was performed using a MgKα spectrometer (JEOL, JPS 9200) in the subsequent circumstances: pass energy = 50 eV (wide-scan) and 30 eV (narrow-scan), Voltage = 10 kV, Current = 20 mA. A PANalytical X-ray diffractometer was used to acquire X-ray diffraction (XRD) spectra with Cu Kα1/2, λα_1_ = 154.060 pm, λα_2_ = 154.439 pm radiation. A “Raman station 400 (Perkin Elmer)” spectrometer was used to acquire the Raman spectra. A FE-SEM (JEOL-6300F, 5 kV) was used to analyze the morphology and structural characteristics of Yb_2_O_3_.CuO@rGO. EDS (JEOL) was used to investigate the elemental composition of the Yb_2_O_3_.CuO@rGO. A JEOL JEM-2100F-UHR field emission apparatus fitted with a Gatan GIF 2001 energy filter and a 1 k-CCD camera was used to capture transmission electron microscopy (TEM) micrographs at 200 kV. Electrochemical measurements were conducted using a Zahner Zennium potentiostat.

### 2.2. Synthesis of CuO, Yb_2_O_3_, Yb_2_O_3_.CuO, and Yb_2_O_3_.CuO@rGO Nanocomposite

To synthesize the CuO, Yb_2_O_3_, Yb_2_O_3_.CuO, and Yb_2_O_3_.CuO@rGO nanocomposites, the following process was followed: firstly, equimolar Cu(NO_3_)_2_ and Yb(NO_3_)_3_ solutions were mixed in a beaker and stirred for half an hour at 70 °C. This mixture was then combined with NaOH and stirred vigorously at 80 °C for 8 h. Afterwards, the ensuing dark precipitate was cleaned with distilled water and ethanol to remove contaminants and the resulting black precipitate was dried at 80 °C. This as-grown Yb_2_O_3_.CuO nanoparticle (NP) was then calcined by heating it for six hours at 500 °C in a furnace. During this synthesis process, the following chemical reactions occurred:

Cu(NO_3_)_2_ + 2NaOH → Cu(OH)_2_ + 2NaCl

Yb(NO_3_)_3_ + 3NaOH → Yb(OH)_3_ + 3NaCl

Cu(OH)_2_ + 2Yb(OH)_3_ → Yb_2_O_3_.CuO + 4H_2_O

Precursors, Yb^3+^ and Cu^2+^ ions are soluble in NaOH solution, where NaOH keeps the pH constant during the reaction and continuously releases OH^−^. The development of the Cu(OH)_2_ nucleus starts when the ionic product of Cu^2+^ and OH^−^ exceeds the K_sp_ value. Similarly, Yb(OH)_3_ was also produced. Cu^2+^ ions easily incorporate themselves into the Yb_2_O_3_ lattice because of the similar ionic radii. On heating, hydroxides decompose to produce respective oxides. Similarly, CuO and Yb_2_O_3_ NPs were also synthesized.

To synthesize the Yb_2_O_3_.CuO@rGO nanocomposite, 0.5 g of Yb_2_O_3_.CuO NPs and 0.025 g of reduced graphene oxide (rGO) were mixed followed by 40 min of sonication in 80 mL distilled water. This resulting mixture was then filtered and had 12 h of drying in an oven at 70 °C.

### 2.3. Glassy Carbon Electrode Modification Using Yb_2_O_3_.CuO@rGO Nanocomposite

Glassy carbon electrodes (GCEs) (diameter = 3 mm; BAS Inc., Sumida-Ku, Japan) were cleaned using a 1 µm diamond past, followed by a 0.05 µm alumina slurry using the commercially available polishing pads. Next, the GCE was fabricated utilizing Yb_2_O_3_.CuO@rGO nanocomposite using a Nafion solution. During the fabrication process, 4.0 mg of Yb_2_O_3_.CuO@rGO was uniformly mixed with 0.05 mL Nafion and 0.45 mL propan-2-ol, and then 2 µL of this suspension was carefully applied to a pre-cleaned GCE and dried at 60 °C for 20 min. Such a fabricated GCE was labeled as the Yb_2_O_3_.CuO@rGO/GCE. Control experiments were also conducted, in which CuO/GCE, Yb_2_O_3_/GCE, rGO/GCE, and Yb_2_O_3_.CuO/GCE were fabricated using similar procedures. The electrochemical investigations of AA (0.5–1744 µM) were carried out in a typical three-electrode electrochemical cell at ambient conditions in 0.1 M PBS (pH 7.0), a Yb_2_O_3_.CuO@rGO/GCE, Ag/AgCl, and a platinum spiral were served as the working, reference, and counter electrodes, respectively.

## 3. Results and Discussion

### 3.1. Characterization of Yb_2_O_3_.CuO@rGO Nanocomposite

**Figure 1:**
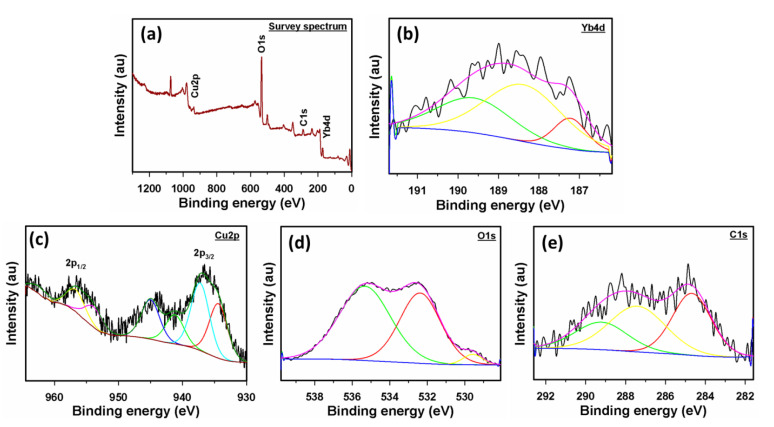
(**a**) Survey XPS spectrum of Yb_2_O_3_.CuO@rGO NC, (**b**) deconvoluted spectra of Yb4d, (**c**) Cu2p_,_ (**d**) O1s, and (**e**) C1s of Yb_2_O_3_.CuO@rGO nanocomposite.

The elemental compositions and structure of Yb_2_O_3_.CuO@rGO were examined using XPS. It is evident from the XPS analysis shown in Figure 1a–e that Yb_2_O_3_.CuO@rGO nanocomposite is composed of Yb, Cu, O, and C atoms only. The Yb4d_5/2_ spectrum has three clearly defined peaks appearing at energies of 187.2, 188.4, and 189.1, which are compatible with Yb4d (Figure 1b) [50]. In the deconvoluted Cu2p spectrum in Figure 1c, there are two peaks at 937.1 and 956.8 eV that may be related to Cu2p_3/2_ and Cu2p_1/2_, respectively [51]. In between these two peaks, there are some satellite peaks that appeared that are also consistent with the literature [52]. Figure 1d shows two peaks from the fine-scan O1s spectra that are associated with the Yb–O and Cu–O bonds, respectively, at 533.3 and 535.2 eV [13]. Three peaks are shown in the fine-scan C1s spectrum in Figure 1e at energies of 284.6, 287.2, and 289.1 eV. The peaks at 284.6 and 287.2 eV may be attributed to C–C and C–O–H bonds, respectively [53], and the remaining peak at 289.1 eV can be correlated to COOH [54].

**Figure 2:**
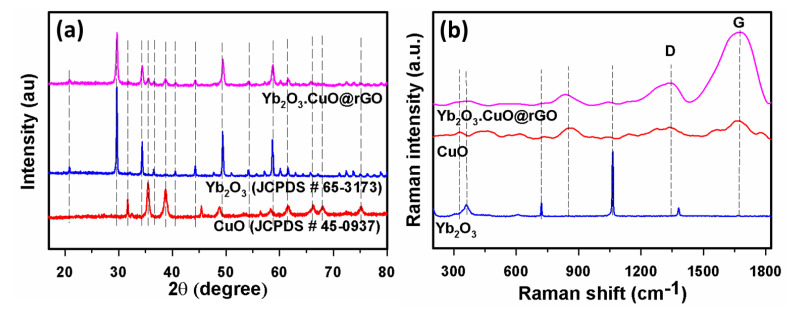
(**a**) XRD patterns and (**b**) Raman spectra of CuO, Yb_2_O_3_, and Yb_2_O_3_.CuO@rGO NC.

XRD patterns in Figure 2a showed diffraction bands at 2θ = 20.80, 29.50, 34.30, 36.50, 40.60, 44.00, 47.50, 49.20, 51.00, 54.10, 57.10, 58.50, 60.00, and 61.50, which are related to the (211), (222), (400), (411), (332), (134), (125), (440), (443), (611), (145), (662), (136), and (444) planes for Yb_2_O_3_ NPs (JCPDS#65-3173), respectively [50]. The diffraction bands at 35.40, 38.60, 48.60, 58.20, 61.60, 66.30, and 68.10 can be related to (002), (111), (−202), (202), (−113), (−311), and (220) planes of CuO NPs ((JCPDS#45-0937), respectively [55]. The Yb_2_O_3_.CuO@rGO contains the rGO peak connected to carbon that is often appearing at 2θ = 24.30 which is correlated to (002) plane [56] but is not easily visible in Figure 2a due to low intensity. However, the presence of carbon in Yb_2_O_3_.CuO@rGO was established by XPS, EDS, SEM, and TEM. Figure 2b shows the Raman spectra, where bands at 359.3, 718, and 1060 cm^−1^ can be related to Yb_2_O_3_, while bands at 328 and 850 cm^−1^ were connected to CuO [57]. The characteristic carbon bands at 1344 and 1676 cm^−1^ are related to the D and G bands of rGO [58].

**Figure 3:**
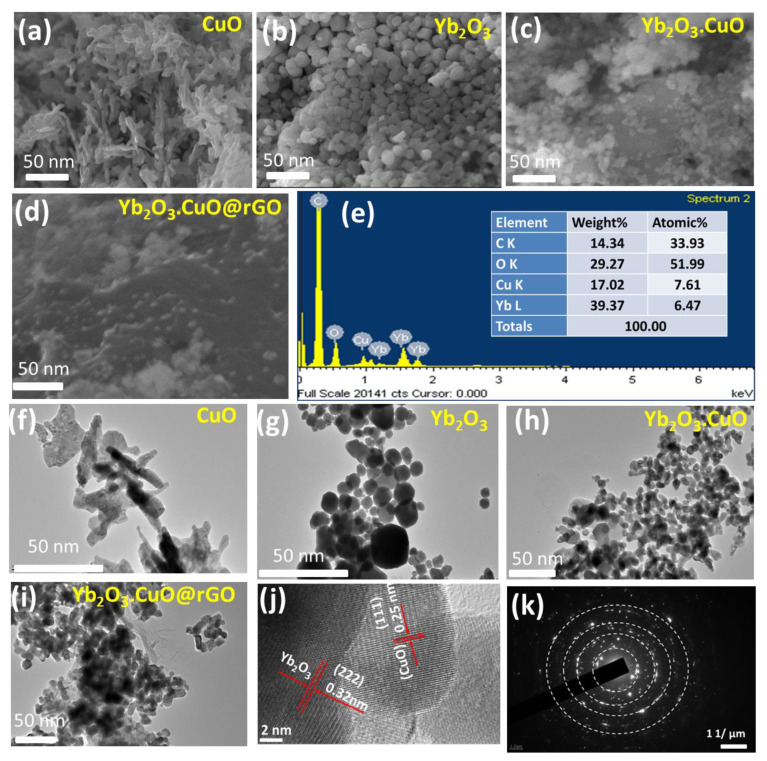
FESEM image: (**a**) CuO, (**b**) Yb_2_O_3_, (**c**) Yb_2_O_3_.CuO, (**d**) Yb_2_O_3_.CuO@rGO, (**e**) EDS spectrum of Yb_2_O_3_.CuO@rGO; TEM micrograph from (**f**) CuO, (**g**) Yb_2_O_3_, (**h**) Yb_2_O_3_.CuO, (**i**) Yb_2_O_3_.CuO@rGO, (**j**) HR-TEM image, and (**k**) SAED patterns of Yb_2_O_3_.CuO@rGO nanocomposite.

FESEM was employed to analyze the morphological and surface structure of CuO, Yb_2_O_3_, Yb_2_O_3_.CuO, and Yb_2_O_3_.CuO@rGO nanocomposite as presented in Figure 3a–d. The Yb_2_O_3_.CuO@rGO nanocomposite was found to be made up of Yb_2_O_3_.CuO composites that were randomly distributed over the graphene sheets. EDS was used to determine the Yb_2_O_3_.CuO@rGO nanocomposite’s elemental composition (Figure 3e), and the results showed that the nanocomposite is exclusively made of Yb, Cu, O, and C with their respective weight percentages as 39.37%, 17.02%, 29.27%, and 14.34%. This elemental composition agrees with the findings of XPS and XRD. A more thorough morphology of CuO, Yb_2_O_3_, Yb_2_O_3_.CuO, and Yb_2_O_3_.CuO@rGO nanocomposite was provided by the TEM images in Figure 3f–i that show a collection of spherical Yb_2_O_3_ and elongated CuO NPs dispersed on sheet-like structures of rGO. Figure 3j presents an HR-TEM image of the Yb_2_O_3_.CuO@rGO nanocomposite and Figure 3k displays the selected area electron diffraction (SAED) patterns, which unequivocally reveal that the composite is polycrystalline.

### 3.2. Ascorbic Acid Sensor Development

#### 3.2.1. Electrochemical Study of Yb_2_O_3_.CuO@rGO/GCE Assembly

**Figure 4:**
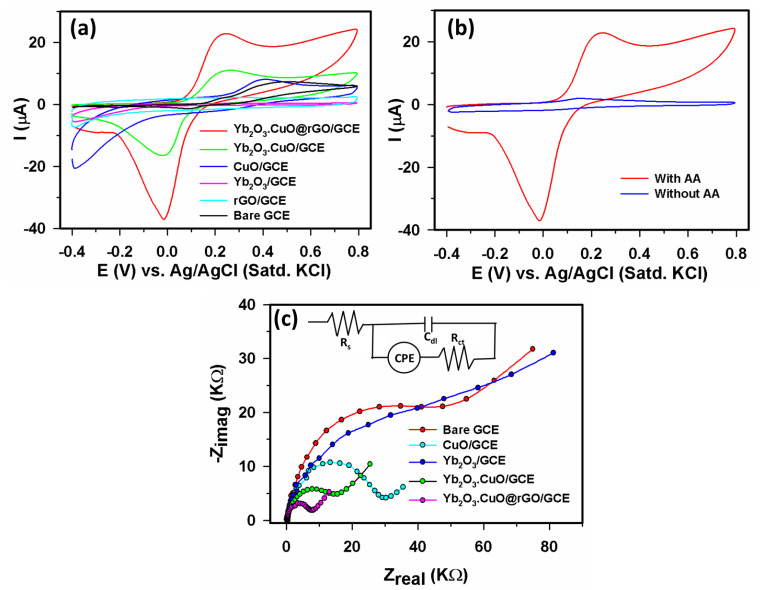
CVs recorded at scan rate 0.05 Vs^−1^ in 0.1 M PBS (pH 7.0) (**a**) CVs from bare GCE, CuO/GCE, Yb_2_O_3_/GCE, rGO/GCE, Yb_2_O_3_.CuO/GCE, Yb_2_O_3_.CuO@rGO/GCE with 40 µM AA, (**b**) CVs from the Yb_2_O_3_.CuO@rGO/GCE with 40 µM AA and without AA, and (**c**) EIS Nyquist plots acquired using various electrodes in 1.0 mM [Fe(CN)_6_]^3−/4−^ in 0.1 M KCl at +0.50 V, at signal amplitude 10 mV, and with frequency ranging from 0.1 Hz to 100 KHz with a relevant equivalent circuit in the inset.

We evaluated the electro-chemical activity of the modified electrodes through cyclic voltammetry (CV) and electrochemical impedance spectroscopy (EIS). Figure 4a illustrates the feeble CV response from a bare GCE in the presence of 40 µM AA at +0.52 V; however, the CuO/GCE and Yb_2_O_3_.CuO/GCE showed enhanced CV outputs at +0.41 V and 0.28 V, respectively. For the Yb_2_O_3_/GCE and rGO/GCE electrodes, no CV response was detected. A significantly improved CV result at a low potential of +0.25 V was obtained from the Yb_2_O_3_.CuO@rGO/GCE. This demonstrates that this Yb_2_O_3_.CuO@rGO/GCE assembly possessed the greatest electrocatalytic performance during AA determination in comparison to other the electrodes shown in Figure 4a. Therefore, we designated the Yb_2_O_3_.CuO@rGO/GCE assembly as an AA sensor in this investigation. Additionally, a definite CV peak was produced for the Yb_2_O_3_.CuO@rGO/GCE sensor with 40 µM AA while, in the absence of AA, no CV response was seen (Figure 4b), further emphasizing the effective electro-chemical properties of Yb_2_O_3_.CuO@rGO/GCE as an AA sensor. Figure 4c displays EIS Nyquist plots of bare GCE, CuO/GCE, Yb_2_O_3_/GCE, Yb_2_O_3_.CuO/GCE, and Yb_2_O_3_.CuO@rGO/GCE, and a relevant equivalent circuit is presented in the inset. The Yb_2_O_3_.CuO@rGO/GCE electrode was found to have the shortest semicircle diameter, which indicates that its charge transfer resistance (Rct = 9.2 kΩ) value is lower than that of other electrodes including bare GCE (75.2 kΩ), CuO/GCE (35.2 kΩ), Yb_2_O_3_/GCE (94.7 kΩ), and Yb_2_O_3_.CuO/GCE (22.9 kΩ), which were acquired through fitting utilizing the EIS Spectrum Analyzer Software. The smallest semicircular diameter of the Yb_2_O_3_.CuO@rGO/GCE electrode suggests that the fabrication process lowered its Rct value. We therefore draw the conclusion that the Yb_2_O_3_.CuO@rGO/GCE electrode provided improved electron transfer performance than the other modified electrodes shown in Figure 4a.

**Figure 5:**
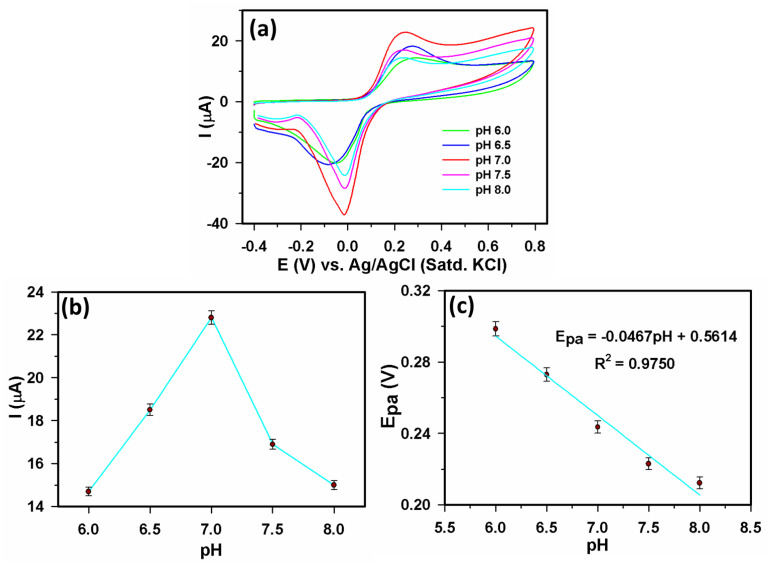
(**a**) CVs recorded using 40 µM AA in 0.1 M PBS at varying pH (6.0–8.0) at 0.05 Vs^−1^ scan rate, (**b**) I_pa_ vs. pH, and (**c**) E_pa_ vs. pH.

We investigated the impact of a pH between 6.0 and 8.0 with 40 µM AA to better understand the electrochemical AA oxidation. Figure 5a,b shows that, for a pH of 6.0 to 7.0, the anodic peak current (I_pa_) value steadily increased and, for a pH 7.0 to 8.0, a declining trend was seen. The extreme I_pa_ was seen at a pH ~ 7.0, as shown in Figure 5b. As a result, pH 7.0 was set as the standard for the remaining tests in this paper. Figure 5c displayed a straight-line plot for anodic peak potential (E_pa_) vs. pH having a regression Equation (1):

E_pa_(V) = 0.5614 − 0.0467pH   (R^2^ = 0.9750) (1)

Figure 5c showed that the gradient of −56 mV per pH unit over the selected pH range is extremely near to the predicted value of −59, demonstrating that the quantity of transported protons and electrons involved in this AA oxidation are equal [13,19].

**Figure 6:**
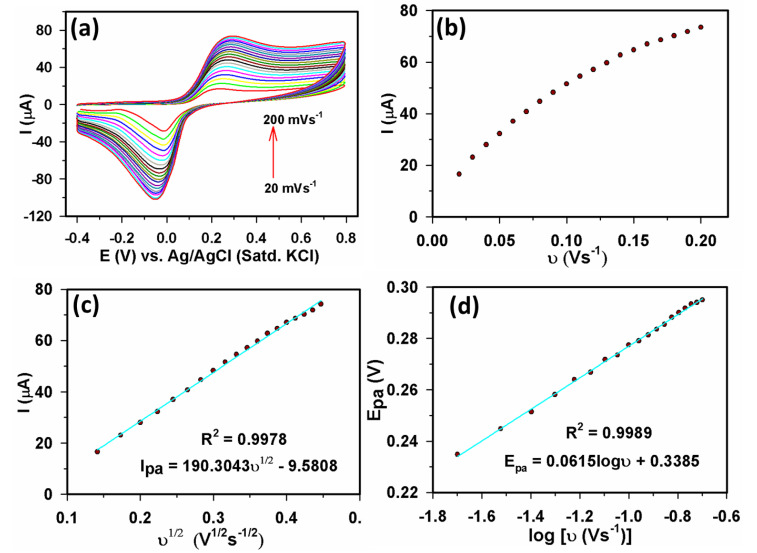
Investigation of scan rate effect of Yb_2_O_3_.CuO@rGO/GCE sensor: (**a**) CVs recorded at different scan rates (20–200 mVs^−1^) with 40 µM AA in 0.1 M PBS (**b**) I_pa_ vs. *v*, (**c**) I_pa_ vs. √v, and (**d**) E_pa_ vs. log(*v*).

Scan rate (*v*) analysis in Figure 6a shows the CVs of 40 µM AA acquired using different scan rates (20–200 mVs^−1^) using a Yb_2_O_3_.CuO@rGO/GCE sensor. The I_pa_ value in Figure 6a was rising as *v* increased, although the E_pa_ value only marginally changed in a positive way. The nonlinear change in I_pa_ vs. *v* in Figure 6b suggested that AA oxidation is not a surface-controlled process [59] while, in Figure 6c, a linear I_pa_ vs. *v*^1/2^ curve was seen, validating a diffusion-controlled process [60] using Equation (2) below.

I_pa_(µA) = 190.3043 *v*^1/2^ (V^1/2^s^−1/2^) − 9.5808  (R^2^ = 0.9978) (2)

Additionally, in Figure 6d, a straight line from E_pa_ vs. log(*v*) plot was seen using Equation (3).

E_pa_ (V) = 0.0615 log[*v* (Vs^−1^)] + 0.3385  (R^2^ = 0.9989) (3)

Figure 6a exhibited that for *v* > 70 mVs^−1^, the value of [E_pa_ − E_pc_]/2 remained essentially unchanged. Hence, at 100 mVs^−1^ scan rate, the [E_pa_ − E_pc_]/2 value assume to be 90.5/nα mV [61], consequently, it was determined that there were 2.29 ≈ 2 transferred electrons (n_α_). Therefore, it is established that AA oxidation at the Yb_2_O_3_.CuO@rGO/GCE surface was a two-electron-transfer system. Overall, the scan rate and pH investigations determined that AA oxidation at the Yb_2_O_3_.CuO@rGO/GCE surface is a combined two-electrons and two-protons reaction, which is consistent with the literature [13].

#### 3.2.2. Sensor Parameters Determination

**Figure 7:**
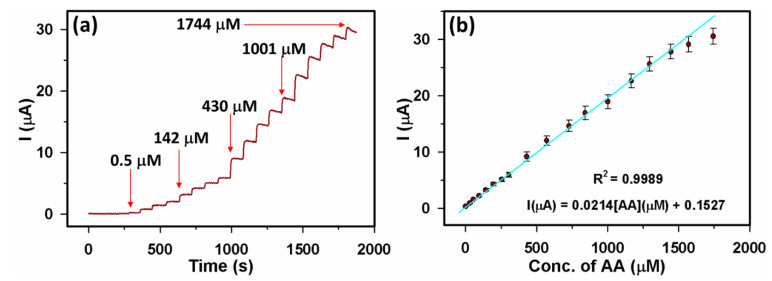
(**a**) Yb_2_O_3_.CuO@rGO/GCE sensor’s amperometric response for AA (0.5–1744 µM) at +0.3 V potential, and (**b**) related calibration plot.

We used amperometry for evaluating the sensor performance of the Yb_2_O_3_.CuO@rGO/GCE sensor. An amperometric response was acquired at +0.3 V after adding AA of varying concentrations (0.5–1744 µM) at consecutive time intervals. Figure 7a displays the amperometric responses achieved from the Yb_2_O_3_.CuO@rGO/GCE sensor for AA additions. Here, the current response in each AA addition increased to around 95% of its maximum current in just 4 s. Figure 7b shows a linear segment of calibration plot for 0.5–1744 µM AA using the Equation (4).

I (μA) = 0.0214 [AA] (µM) + 0.1527  (R^2^ = 0.9989) (4)

As a result, the Yb_2_O_3_.CuO@rGO/GCE sensor’s linear detection range (LDR) was determined to be 0.5–1571 μM. Additionally, the Yb_2_O_3_.CuO@rGO/GCE sensor’s estimated sensitivity value was found to be 0.4341 μAμM^−1^cm^−2^ and limit of detection (LOD) and limit of quantification (LOQ) were determined to be ~0.062 μM (S/N = 3) and 0.1887 µM, respectively. The sensitivity was calculated using the equation, sensitivity = S/A_eff_ [62], where A_eff_ stands for the surface area of the modified electrode (0.0493 cm^2^), as provided in the electronic Supplementary Materials of Figure S1 [19,63,64]. The equations were used to calculate LOD and LOQ are LOD = 3.3(S_b_/S) and LOQ = 10(S_b_/S), respectively [65]; here, S_b_ (0.000403) stands for relative standard deviation (RSD) related to five blank responses, and S stands for calibration curve’s slope.

The electrocatalytic performance is dependent on two variables: (i) increase in I_pa_ and (ii) decreased E_pa_. Hence, attempts have been made to improve the electrocatalytic activity of GCEs by fabricating them using Yb_2_O_3_.CuO@rGO NC. The achieved results showed that the Yb_2_O_3_.CuO@rGO/GCE sensor successfully satisfied both of the aforementioned requirements. Figure 4a showed a substantial negative shift of E_pa_ and a significant increase in I_pa_ from the Yb_2_O_3_.CuO@rGO/GCE sensor compared to other electrodes used in this study. We achieved about a three-fold I_pa_ from the Yb_2_O_3_.CuO@rGO/GCE compared to a bare GCE during AA oxidation.

#### 3.2.3. Selectivity, Repeatability, Reproducibility, and Stability

**Figure 8:**
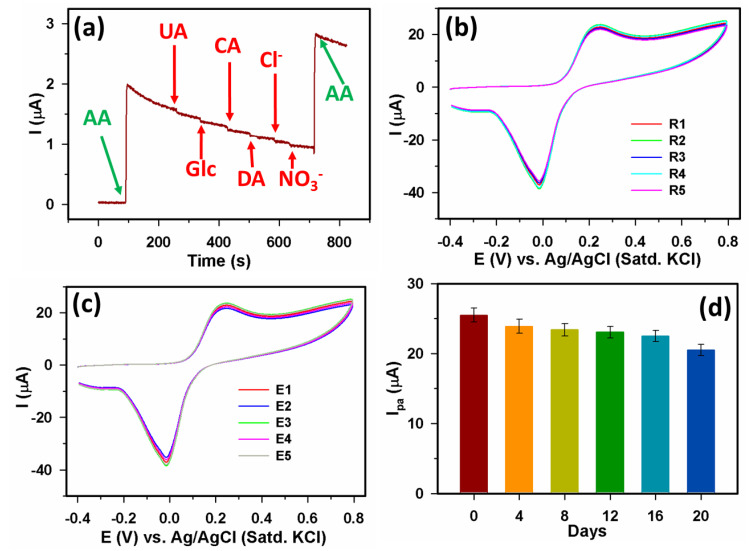
(**a**) Amperometric (*i–t*) response at +0.3 V from Yb_2_O_3_.CuO@rGO/GCE sensor upon successive additions of 90 µM of AA, UA, Glc, CA, DA, Cl^−^, NO_3_^−^, and AA, (**b**) repeatability, (**c**) reproducibility, and (**d**) stability investigations.

To test the Yb_2_O_3_.CuO@rGO/GCE sensor’s selectivity, we used common interfering chemicals such as uric acid (UA), glucose (Glc), citric acid (CA), dopamine (DA), Cl^-^ ions, and NO_3_^-^ ions. Here, 90 μM AA and an equal concentration of each interfering chemical were used to record the amperometric response (Figure 8a). While AA addition generated a significant amperometric response, no response was observed for the interfering chemicals. This confirms the selectivity of the Yb_2_O_3_.CuO@rGO/GCE assembly during the AA detection. Furthermore, the various sensor characteristics of Yb_2_O_3_.CuO@rGO/GCE were also investigated using CV with 40 M AA. A freshly fabricated Yb_2_O_3_.CuO@rGO/GCE assembly was employed to measure 40 M AA for the repeatability study shown in Figure 8b. Five runs with a 4.2% RSD and with nearly similar CV responses showed good repeatability. Figure 8c showed the reproducibility study of the Yb_2_O_3_.CuO@rGO/GCE assembly that used five newly modified Yb_2_O_3_.CuO@rGO/GCE electrodes (E1–E5). The Ipa variations in CV responses revealed a 4.7% RSD, demonstrating remarkable reproducibility. In addition, we recorded CV responses every fourth day for a newly modified Yb_2_O_3_.CuO@rGO/GCE sensor to assess its stability while keeping it at room temperature. Figure 8d displays the stability investigation bar graph. It demonstrates that the I_pa_ value in CVs was retained at approximately 81% of its initial value after being stored for 20 days at ambient conditions and that the Yb_2_O_3_.CuO@rGO/GCE sensor surface remained undamaged.

When the AA molecule touches the Yb_2_O_3_.CuO@rGO surface, an electro-oxidation reaction occurs. AA molecules release electrons to the conduction-band of the Yb_2_O_3_.CuO@rGO nanocomposite that ultimately enhance the conductivity of the Yb_2_O_3_.CuO@rGO/GCE sensor and, hence, an enhanced CV response can be obtained. In comparison to other AA sensors, the Yb_2_O_3_.CuO@rGO/GCE sensor demonstrated a greater sensitivity for AA detection (Table 1) [13,17,39,65,66,67,68,69,70,71,72,73].

**Scheme 1:**
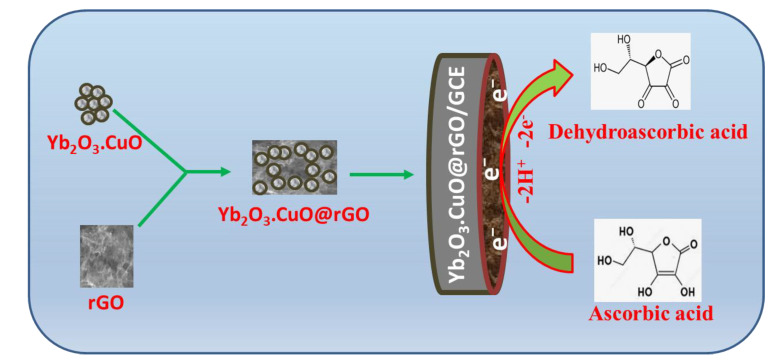
Schematic representation for Yb_2_O_3_.CuO@rGO/GCE−based ascorbic acid sensor.

Considering the experimental findings stated above, we may say that AA oxidation at the Yb_2_O_3_.CuO@rGO NC is a combined two-electrons and two-protons transfer reaction and, in this AA oxidation, the Yb_2_O_3_.CuO@rGO NC is exceedingly active. The Yb_2_O_3_.CuO@rGO/GCE sensor’s appropriateness in detecting AA can be attributed to the effective electrode-analyte interaction. Scheme 1 shows a concise model for electrochemical AA oxidation at this novel Yb_2_O_3_.CuO@rGO/GCE sensor.

### 3.3. Analyses of Real Samples: AA Detection from Blood Serum and Vitamin C Tablet

The developed suggested Yb_2_O_3_.CuO@rGO/GCE sensor’s efficacy was tested by measuring AA in blood serums and vitamin C tablets utilizing the standard addition method. Firstly, we measured the Yb_2_O_3_.CuO@rGO/GCE sensor’s (*i–t*) response at +0.3 V in 10 mL PBS with 200 µL of undiluted blood serum (BS1) and then three repeated injections of 50 µL 0.01 M AA. Such processes were carried out three times under the same circumstances. Next, we performed the same standard addition procedure using the second blood serum (BS2). Furthermore, we used a dissolved Vitamin C 1000 tablet (Vit-C) from Dallah Pharma Factory, KSA as the real sample, as in our previous report [13]. Finally, we repeated the whole standard addition process using 100 µL of Vitamin C and then three repeated injections of 100 µL 0.01 M AA. Table 2 summarizes the outcomes of the real sample investigations. These results indicate that, with approximately 100% quantitative recovery, this novel Yb_2_O_3_.CuO@rGO/GCE sensor can be utilized to precisely assess the presence of AA in real samples. Additionally, the measured level of AA in blood serums is within AA levels typically found in adults (28.5–85.2 µM) [7] and, for the Vitamin C tablets, the calculated AA amount was 98.1% of the manufacturer’s specification, confirming that the newly-developed Yb_2_O_3_.CuO@rGO/GCE sensor is appropriately validated.

## 4. Conclusions

Herein, we successfully synthesized and characterized the Yb_2_O_3_.CuO@rGO nanocomposite. This nanocomposite material was then used to design a sensitive, selective, and reusable electrochemical AA sensor. This AA sensor was developed by a facile technique and is able to measure both high and low levels of AA because of its broad linear dynamic range and high sensitivity. Additionally, this AA sensor demonstrated a minimal interference effect, a fast response time, a reasonable limit of detection, excellent stability, reproducibility, and repeatability. These features make it a promising tool for detecting AA. To further validate the Yb_2_O_3_.CuO@rGO/GCE sensor’s accuracy, it was tested utilizing blood serums and vitamin C tablets, and the results were consistent and encouraging. Overall, the method of sensor fabrication presented in this study offers a promising platform for developing a highly efficient AA sensor in the future.

## Figures and Tables

**Table 1:** Comparative ascorbic acid sensor performance employing various electrodes.

Electrode	Technique	LDR/μM	LOD/µM	Sensitivity/μAμM^−1^cm^−2^	Applied Potential/V	Ref.
PSi-MC/GCE	Amp	0.5–2473	0.03	0.1982	+0.7	[13]
Poly(Py-oPD)/PGE	SWV	1–1000	0.026	-	-	[17]
GO-IL/GCE	Amp	10–4000	3.33	-	+0.8	[66]
DMA/GCE	Amp	25–1650	-	0.178	+0.35	[67]
PoPDoAP/GCE	DPV	100–1000	36.4	0.0306 μAμM^−1^	-	[68]
NFG/Ag/PANI	Amp	10–11,460	8.0	-	+1.2	[69]
PG/GCE	Amp	9.0–2314	6.45	0.0667 μAμM^−1^	−0.01	[70]
ZnO/GCE	Amp	1–800	0.27	0.1156 μAμM^−1^	+0.36	[71]
ERGO/GCE	DPV	500–2000	150	0.0054 μAμM^−1^	-	[72]
PMES/RGO/GCE	DPV	30–100	0.43	-	-	[73]
NPG	Amp	10–1100	2.0	0.0021 μAμM^−1^	+0.3	[74]
GCE/Au@Pd-RGO	DPV	0.01–100	0.002	-	-	[75]
Yb_2_O_3_.CuO@rGO/GCE	Amp	0.5–1571	0.062	0.4341	+0.25	This work

PSi-MC = porous silicon-mesoporous carbon; Amp = Amperometry, DMA = N,N Dimethylaniline, GO-IL = Graphene oxide–Ionic liquid, PoPDoAP = Poly(o-phenylenediamineco-o-aminophenol), NFG = nanoparticles grafted functionalized graphene, PG *=* pristine graphene, Poly(Py-oPD)/PGE = pencil graphite electrode modified with a molecularly imprinted copolymer of pyrrole and o-phenylenediamine, PMES = Poly(2-(N-morpholine)ethane sulfonic acid), ERGO = electrochemically reduced graphene oxide, and NPG = nanoporous gold.

**Table 2:** AA Detection from commercial vitamin C tablets and blood serums (BS1 and BS2) using the Yb_2_O_3_.CuO@rGO/GCE sensor.

Real Samples	Added Std. AA (µM)	Total AA Measured (µM)	AA Measured in Real Samples (µM)	Recovery (%)	RSD (%) (*n* = 3)
BS1	48.8 97.6	96.2	46.2	102.4	4.52
		147.4		103.7	
BS2	48.8	88.1	36.5	105.7	4.13
	97.6	137.0		103.0	
Vit-C	98.0	176.6	82.4	96.1	4.37
	194.2	271.1		97.2	

## Data Availability

Data will be available upon request.

## Supplementary Materials

The following supporting information can be downloaded at: https://www.mdpi.com/article/10.3390/bios13060588/s1, Figure S1: (a) CVs recorded with 5 mM [Fe(CN)_6_]^3−/4−^ in 0.1 M KCl using the Yb_2_O_3_.CuO@rGO/GCE assembly for scan rates ranging from 20 to 120 mVs^−1^ (b) I_pa_ vs. ν^1/2^.
