# Supplementary material for: Low Overpotential Amperometric Sensor Using Yb2O3.CuO@rGO Nanocomposite for Sensitive Detection of Ascorbic Acid in Real Samples
Source: Biosensors (Basel). 2023 May 29;13(6):588. doi: 10.3390/bios13060588 (PMC10295996; doi:10.3390/bios13060588)
Supplement: Supplementary file 1 [file biosensors-13-00588-s001.zip › biosensors-2361960-supplementary.pdf]

biosensors

Supporting Information

Low Overpotential Amperometric Sensor using Yb_2O_3.CuO@rGO Nanocomposite for Sensitive Detection of Ascorbic Acid in Real Samples

Jahir Ahmed[1,2], Mohd Faisal[1,2], Jari S. Algethami[1,2], Mabkhoot Alsaiari[1,3], Saeed A. Alsareii[1,4] and Farid A. Harraz[1,3,*]

1 Promising Centre for Sensors and Electronic Devices (PCSED), Advanced Materials and Nano-Research Centre, Najran University, Najran 11001, Saudi Arabia
2 Department of Chemistry, Faculty of Science and Arts, Najran University, Najran 11001, Saudi Arabia
3 Department of Chemistry, Faculty of Science and Arts at Sharurah, Najran University, Sharurah 68342, Saudi Arabia
4 Department of Surgery, College of Medicine, Najran University, Najran 11001, Saudi Arabia
* Correspondence: faharraz@nu.edu.sa

Calculation of effective electrode surface area of the fabricated electrode

We recorded the CVs for 5 mM $[Fe(CN)_6]^{3-/4-}$ in 0.1 M KCl using the Yb_2O_3.CuO@rGO/GCE assembly for scan rates ranging from 20 to 120 mVs^{-1} (Figure. S1a).

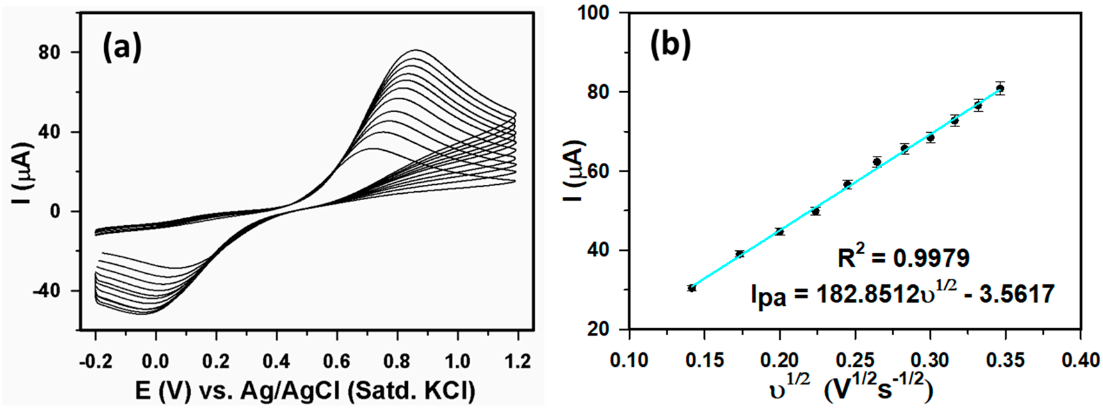

Figure S1. (a) CVs recorded with 5 mM $[Fe(CN)_6]^{3-/4-}$ in 0.1 M KCl using the Yb_2O_3.CuO@rGO/GCE assembly for scan rates ranging from 20 to 120 mVs^{-1} and (b) I_{pa} vs. $\nu^{1/2}$.

From the slope (1.8285×10^{-4} $AV^{-1/2}s^{1/2}$) of the I_{pa} vs. $\nu^{1/2}$ plot (Figure. S1b), using the Randles–Sevcik equation, $I_{pa} = (2.69 \times 10^5)\, n^{3/2} A_{eff} D^{1/2} C_o \nu^{1/2}$, we used the following equation: $1.8285 \times 10^{-4} = (2.69 \times 10^5)\, n^{3/2} A_{eff} D^{1/2} C_o$.

In the above equation, using the respective values as stated in the manuscript, mentioned here again as n = 1 is the stoichiometric electron number in this electrode reaction; A_{eff} represents the electrode area in cm^2, $D = 7.6 \times 10^{-6}$ cm^2s^{-1} represents the diffusion-coefficient of $[Fe(CN)_6]^{3-/4-}$ [1,2], and $C_o = 5 \times 10^{-6}$ $molcm^{-3}$ is the $[Fe(CN)_6]^{3-/4-}$ solution concentration.

We have accordingly calculated the effective surface area of the modified electrode as follows:

$A_{eff} = (1.8285\times10^{-4}) / [2.69\times10^5 \times 1^{3/2}\times (7.6\times10^{-6})^{1/2}\times (5.0\times10^{-6})] = 0.0493$ cm^2

Similarly, we have calculated the effective surface area of a bare GCE (0.0329 cm^2).

References

1. Ahmed, J.; Rashed, M.A.; Faisal, M.; Harraz, F.A.; Jalalah, M.; Alsareii, S.A. Novel SWCNTs-Mesoporous Silicon Nanocomposite as Efficient Non-Enzymatic Glucose Biosensor. *Appl. Surf. Sci.* **2021**, *552*, 149477, doi:10.1016/j.apsusc.2021.149477.
2. Ahmed, J.; Faisal, M.; Jalalah, M.; Alsaiari, M.; Alsareii, S.A.; Harraz, F.A. An Efficient Amperometric Catechol Sensor Based on Novel Polypyrrole-Carbon Black Doped α-Fe_2O_3 Nanocomposite. *Colloids Surfaces A Physicochem. Eng. Asp.* **2021**, *619*, 126469. https://doi.org/10.1016/j.colsurfa.2021.126469.
